# Deep Learning-Based Computer-Aided Detection System for Automated Treatment Response Assessment of Brain Metastases on 3D MRI

**DOI:** 10.3389/fonc.2021.739639

**Published:** 2021-10-27

**Authors:** Jungheum Cho, Young Jae Kim, Leonard Sunwoo, Gi Pyo Lee, Toan Quang Nguyen, Se Jin Cho, Sung Hyun Baik, Yun Jung Bae, Byung Se Choi, Cheolkyu Jung, Chul-Ho Sohn, Jung-Ho Han, Chae-Yong Kim, Kwang Gi Kim, Jae Hyoung Kim

**Affiliations:** ^1^Department of Radiology, Seoul National University Bundang Hospital, Seongnam, South Korea; ^2^Department of Biomedical Engineering, Gachon University Gil Medical Center, Incheon, South Korea; ^3^Center for Artificial Intelligence in Healthcare, Seoul National University Bundang Hospital, Seongnam, South Korea; ^4^Department of Radiology, Vietnam National Cancer Hospital, Hanoi, Vietnam; ^5^Department of Radiology, Seoul National University Hospital, Seoul, South Korea; ^6^Department of Neurosurgery, Seoul National University Bundang Hospital, Seongnam, South Korea

**Keywords:** brain metastasis, computer-aided detection, machine learning, deep learning, Response Assessment in Neuro-Oncology Brain Metastases (RANO-BM)

## Abstract

**Background:**

Although accurate treatment response assessment for brain metastases (BMs) is crucial, it is highly labor intensive. This retrospective study aimed to develop a computer-aided detection (CAD) system for automated BM detection and treatment response evaluation using deep learning.

**Methods:**

We included 214 consecutive MRI examinations of 147 patients with BM obtained between January 2015 and August 2016. These were divided into the training (174 MR images from 127 patients) and test datasets according to temporal separation (temporal test set #1; 40 MR images from 20 patients). For external validation, 24 patients with BM and 11 patients without BM from other institutions were included (geographic test set). In addition, we included 12 MRIs from BM patients obtained between August 2017 and March 2020 (temporal test set #2). Detection sensitivity, dice similarity coefficient (DSC) for segmentation, and agreements in one-dimensional and volumetric Response Assessment in Neuro-Oncology Brain Metastases (RANO-BM) criteria between CAD and radiologists were assessed.

**Results:**

In the temporal test set #1, the sensitivity was 75.1% (95% confidence interval [CI]: 69.6%, 79.9%), mean DSC was 0.69 ± 0.22, and false-positive (FP) rate per scan was 0.8 for BM ≥ 5 mm. Agreements in the RANO-BM criteria were moderate (**κ**, 0.52) and substantial (**κ**, 0.68) for one-dimensional and volumetric, respectively. In the geographic test set, sensitivity was 87.7% (95% CI: 77.2%, 94.5%), mean DSC was 0.68 ± 0.20, and FP rate per scan was 1.9 for BM ≥ 5 mm. In the temporal test set #2, sensitivity was 94.7% (95% CI: 74.0%, 99.9%), mean DSC was 0.82 ± 0.20, and FP per scan was 0.5 (6/12) for BM ≥ 5 mm.

**Conclusions:**

Our CAD showed potential for automated treatment response assessment of BM ≥ 5 mm.

## Introduction

Brain metastases (BMs) are the most common brain tumors in adults (1, 2). Increasing evidence suggests that stereotactic radiosurgery can be safely used for patients with up to 10 BM nodules (3, 4). Thus, accurate determination of the number, size, and location of metastatic lesions on brain imaging has become crucial for selecting the most appropriate treatment method. The introduction of three-dimensional (3D) sequences in MRI, which allows the acquisition of thin-section thickness images in a reasonable time, has significantly enhanced the sensitivity of BM detection, particularly for small nodules (5, 6). However, this is highly labor intensive and time consuming for radiologists owing to the high number of images, which could account for as many as several hundred images for a single MRI study.

Previous studies found that computer-aided diagnosis system (CAD) increases the sensitivity of detecting lesions in the brain (7–11), breast (12, 13), lung (14, 15), and colon (16). With several milestones (17–20), deep learning (DL) has seen a sudden increase in interest and applications across the field of medical imaging. A few DL approaches based on semantic segmentation using fully convolutional networks have been proposed for BM identification with MRI (21–23). Zhou et al. (24) demonstrated that DL-CAD may assist radiologists in the detection of BM, with limited false-positive (FP) findings. Ardila et al. (25) also reported the possibility of end-to-end lung screening through DL-CAD.

The Response Assessment in Neuro-Oncology Brain Metastases (RANO-BM) criteria (26) stipulates that the sum of the longest diameter of up to five target lesions should be compared between two studies to assess the treatment response of BM, which is also considerably tedious and time-consuming. A recent study showed that semi-automated size measurements of BM could aid in reducing the interobserver variability and assessing the treatment response (27). Thus, this study aimed to develop a DL-CAD for automated BM detection and treatment response evaluation on MRI.

## Materials and Methods

This retrospective study adhered to the relevant reporting guidelines (28–30). The patient information in MRI Digital Imaging and Communications in Medicine files was anonymized and de-identified prior to analysis. The institutional review boards approved the study.

### MRI Examination

At Seoul National University Bundang Hospital (SNUBH), a tertiary hospital, MRI examinations were performed using a 1.5-T (Intera, Philips Healthcare, Best, the Netherlands) or 3.0-T (Achieva or Ingenia, Philips Healthcare) MR scanner with an 8- or 32- channel head coil. The MRI parameters for 3D gradient-echo sequence (GRE) were as follows: field of view (FOV), 240 × 240 mm^2^; acquisition matrix, 240 × 240; slice thickness, 1 mm; number of excitations, 1; repetition time (TR), 8–10.6 msec; echo time (TE), 3.7–5.7 msec; and flip angle, 8°. For contrast enhancement, gadobutrol (Gadovist^®^, Bayer Schering Pharma AG, Berlin, Germany; 0.1 mmol/kg) was intravenously injected.

At Seoul National University Hospital (SNUH), MRI examinations were performed using a 3.0-T (Verio, Siemens Healthcare, Erlangen, Germany, or Discovery 750w, GE Healthcare, Milwaukee, WI) MR scanner. The MRI parameters for the 3D GRE were as follows: FOV, 250 × 250 mm^2^; acquisition matrix, 256 × 256; slice thickness, 1 mm; number of excitations, 1; TR, 1500 msec; TE, 1.9 msec; inversion time, 900 msec; partition, 176; and flip angle, 9°. For contrast enhancement, gadobenate dimeglumine (MultiHance, Bracco Diagnostics, Princeton, NJ; 0.1 mmol/kg) was injected intravenously as a bolus.

While the DL-CAD system analyzed only the 3D GRE contrast-enhanced T1-weighted imaging (T1WI), reviewers also assessed other imaging sequences, including pre-contrast T1WI, T2-weighted images, and fluid-attenuated inversion recovery images, in the routine protocol.

### MRI Analysis

A total of 1710 BM nodules were identified from the SNUBH data (8.0 BM nodules per patient). For the training set, 1298 nodules from 147 MRI examinations in 127 patients were used. For the testing set, 200 nodules on initial MRI and 212 nodules on follow-up MRI in 20 patients were used. The longest diameter of each BM on the axial plane was measured. In the training set, the median BM size was 6.5 mm (interquartile range [IQR], 4.8–9.7 mm). In total, 374 and 924 BMs measured < 5 mm and ≥ 5 mm, respectively. In the temporal test set, the median BM size was 6.0 mm (IQR, 4.1–9.2 mm), and 147 and 265 BMs measured < 5 mm and ≥ 5 mm, respectively. A total of 87 BM in 24 patients were identified from the geographic test set. The median BM size was 7.3 mm (IQR, 4.5–18.0 mm). There were 65 BM lesions that measured ≥ 5 mm. The ground truth for treatment response according to the RANO-BM criteria was assessed by two neuroradiologists (L.S. and B.S.C., with 11 and 22 years of clinical experience, respectively) by consensus as complete response, partial response, stable disease, and progressive disease. Although the RANO-BM criteria defines measurable disease as lesions with a long diameter of ≥ 10 mm, we opted to use the size threshold of measurable disease as 5 mm so that we can include smaller BM nodules. Such modification of the criteria was suggested by the RANO-BM working group only when brain MRI with 1-mm slice thickness and no gap were used (26). The presence of any new lesion, regardless of its size, was assessed as progressive disease. In addition to the conventional criteria using one-dimensional measurement of the longest diameter of BM, we assessed volumetric response using the modified criteria suggested by Oft et al. (31). The regions-of-interest in all BM nodules were carefully drawn along the enhancing tumor margin by an experienced radiologist (T.Q.N., with 11 years of clinical experience) using an in-house developed software.

### Development of the CAD Software

**Figure 1:**
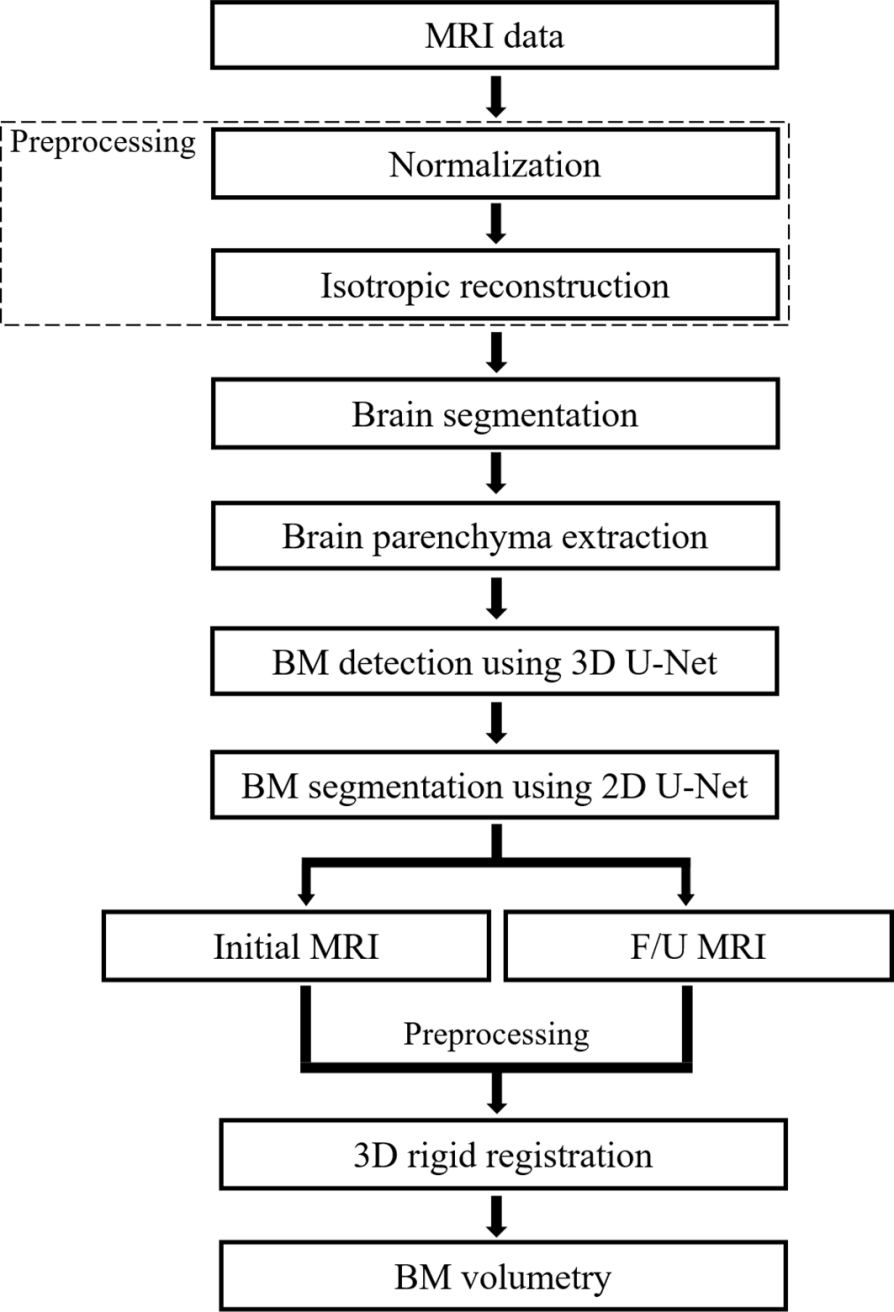
Flowchart of the proposed deep learning-based computer-aided detection system. F/U, follow-up; BM, brain metastasis.

The algorithms of the CAD system were classified into pre-processing, brain segmentation, BM detection, BM segmentation, and BM volumetry. An IBM Power System AC922 8335-GTH (IBM, Armonk, NY, USA) server equipped with four NVIDIA Tesla V100-SXM2 16GB (NVIDIA, Santa Clara, CA, USA) graphics processing units was used for DL. DL training was conducted using Python 2.7.6 and the Keras 2.1.5 framework with a TensorFlow backend in the Ubuntu 14.04 operating system. The programs used in the experiment were Microsoft Visual Studio (Version 2010, Microsoft, Redmond, WA, USA), ITK (Insight Segmentation and Registration Toolkit, Kitware Inc., NY, USA), and VTK (Visualization Toolkit, Kitware Inc., NY, USA). **Figure 1** shows the flowchart of the algorithm that our study proposes.

The signal intensity of each voxel on MRI varies based on the scan parameters. To solve this problem, we normalized the image by resampling the signal of the image excluding the background to a range from 0 to 1 based on the signal intensity of the position manually selected in the gray matter. Then, we limited the application range of the BM detection algorithm to segmented brain regions by automatically cropping the brain regions from the MR image using a DL-based approach. This prevented the CAD from rendering potential false detections that can occur outside the brain regions. Specifically, the 2D U-Net architecture (32), which uses DenseNet201 pre-trained with ImageNet database as encoder (33), was utilized. For model training, we used 3388 MR images that were manually drawn for the brain regions. This was followed by data augmentation using flip, rotation, parallel translation, and scale adjustment (34).

BM has a relatively small size compared to the entire brain area. Therefore, it is difficult to accurately segment the BM in the entire brain region. To solve this problem, we used two DL models. First, we detected the location of the BM, and second, we performed fine segmentation by increasing the size of the detected BM.

The training set was divided into the training and validation datasets using a ratio of approximately 90%:10% (1175:123). We used a 3D U-Net architecture based on the Dice loss function for detecting a relatively small BM compared to the brain (35–38). A 3D structure is advantageous over a 2D structure for recognizing the edges of BM. Furthermore, the Dice loss methodology provides improved detection results for class imbalance and weak boundaries.

The image data were resampled to have a size of 192 × 192 × 192 pixels, i.e., identical along the x, y, and z axes. In addition, the ratio between the axes of the original data was maintained constant *via* zero padding. For the hyperparameters of learning, the Adam activation function was used along with the Dice loss function, and the Epoch, batch size, and learning rate were set to be 300, 1, and 1×10^−3^, respectively. For cases in which the Epoch was between 100 and 250, the learning rate was set to be 1×10^−4^, whereas for cases in which the Epoch was over 250, the learning rate was set to be 1×10^−5^ to lower the learning rate as the learning progresses. The bounding box was calculated based on the information on segmented BM regions *via* the 3D U-Net architecture and used as a BM location.

**Figure 2:**
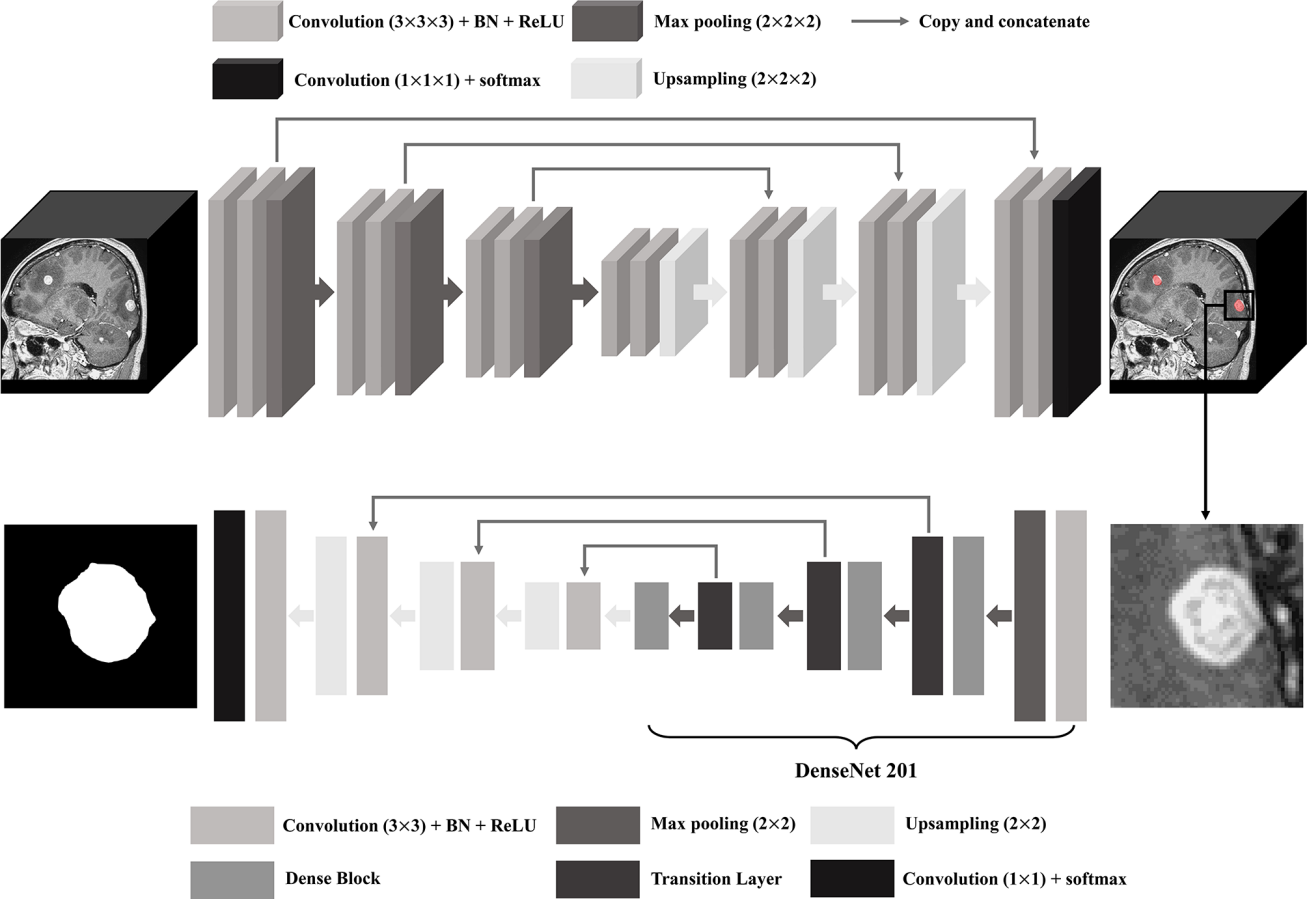
U-Net architecture for brain metastasis (BM) detection and segmentation. BN, batch normalization; ReLU, rectified linear unit.

For BM segmentation, we used the 2D U-Net architecture that uses DenseNet201 pre-trained with ImageNet database as an encoder (32, 33). After cutting off the location of the bounding box circumjacent to BM detected by the 3D U-Net, MR image was resampled to have a size of 512 × 512 pixels so that each BM could fit in the image. The training and testing dataset in the DL model consisted of the same patients and the same number of BM as that in the BM detection using data augmentation by 16 times. The schematic U-Net architecture used for the detection and segmentation of the BM is illustrated in **Figure 2**.

The results segmented by DL were labeled, and the volume for each BM was measured. The volume was measured by calculating the number of pixels included in each segmented BM and multiplying the spacing of x and y axes by the slice thickness.

To match the same BM in the initial and follow-up MRI, we used the 3D rigid registration methodology provided by the ITK library (39, 40). The rigid registration methodology repeats the adjustment and comparison of three parameters, namely, rotation, translation, and scale, while maintaining the morphological structure of a patient in the initial and follow-up images and finds the location where the similarity of these images is the highest. Because the patient in the two images subjected to adjustment was the same, good results were obtained by using only rigid registration. After the adjustment, we could compare the volumes of BM matching in the initial and follow-up MRI.

### Statistical Analysis

We evaluated our CAD in three ways. First, we assessed detection sensitivity, DSC for segmentation, and number of FP nodules per scan in the temporal test set. Second, the success rate of registration on serial MRIs was calculated in the temporal test set. Finally, the one-dimensional and volumetric agreements of the RANO-BM criteria (26) between CAD and the ground truth were calculated using weighted kappa in the temporal test set. Agreement between the two assessments was categorized as poor (κ < 0), slight (κ, 0–0.20), fair (κ, 0.21–0.40), moderate (κ, 0.41–0.60), substantial (κ, 0.61–0.80), and almost perfect (κ > 0.80).

For external validation of CAD using the geographic test set and another temporal test set, the sensitivity and FP per scan were assessed. All statistical analyses were performed with MedCalc (version 19.7; MedCalc Software, Mariakerke, Belgium).

## Results

### Patient Characteristics

From January 2015 to August 2016, 1904 consecutive patients who had a confirmed systemic malignancy and underwent MRI using a “BM work-up” protocol were selected from the radiology database of SNUBH. Patients with a history of primary brain cancers were excluded. Data from January 2015 through March 2016 were collected and labeled according to the previous study (7). Two radiologists (L.S. and J.C., with 11 and 5 years of clinical experience, respectively) reviewed the data from April 2016 through August 2016. All the radiologists had access to the patients’ histories and follow-up imaging studies and determined the reference standard of BM nodules based on consensus.

In total, 1757 patients were excluded based on the following criteria: [1] presence of metastasis involving the bone, dura, or skin or suspicious lesions for leptomeningeal seeding (n = 177), [2] presence of other pathological conditions, such as meningioma, vestibular schwannoma, pituitary adenoma, cavernous malformation, or hemorrhagic infarction (n = 72), [3] presence of equivocal nodule(s) determined to be BM (n = 104), [4] presence of excessive artifacts or poor image quality (n = 31), [5] presence of more than 50 metastatic nodules (n = 32), and [6] absence of BM (n = 1341). Among patients who underwent MRI after April 2016, only those, whose serial MRIs were available, were included in the evaluation of treatment response.

Finally, we included 214 consecutive MRI examinations with post-contrast 3D T1-weighted images conducted between January 2015 and August 2016 in 147 patients with BM (74 women and 73 men; median age, 62 ± 12 years). These were divided into the training dataset (174 MRIs from 127 patients) and the internal test dataset according to temporal separation (hereafter denoted as the temporal test set #1) (40 MRIs from 20 patients). The temporal test set #1 included 12 men and 8 women (mean age ± standard deviation, 63 ± 13 years). The primary malignancies in the temporal test set were lung cancer and breast cancer in 17 and 3 patients, respectively. All patients in the temporal test set #1 had two serial MRIs. The study includes 110 MRIs from 110 patients who had been included in a previous study (7), all of which are included in the training set of the current study. None of the training set cases were included in the test set.

For external validation, we randomly selected 35 patients (19 men; age, 61 ± 12 years) who underwent “BM work-up” MRI at SNUH between May 2014 and March 2015 (hereafter denoted as the geographic test set). In the geographic test set, there were 24 patients with BM and 11 patients without BM. With respect to the type of cancer diagnosis in patients with BM, 18, 2, 2, 1, and 1 patient had lung cancer, breast cancer, melanoma, ovarian cancer, and papillary thyroid carcinoma, respectively. Two experienced neuroradiologists (L.S. and C.H.S., with 11 and 32 years of clinical experience, respectively) reviewed the brain MRI and determined BM based on consensus. We also randomly selected 12 patients (6 men; age, 67 ± 12 years) who underwent “BM work-up” MRI in SNUBH between August 2017 and March 2020 (hereafter denoted as the temporal test set #2). Two experienced neuroradiologists (L.S. and S.J.C., with 11 and 9 years of clinical experience, respectively) reviewed the brain MRI and determined BM based on consensus. For indeterminate lesions even after consensus, we used available follow-up MRI for adjudication. The clinicodemographic characteristics of the included patients are summarized in **Table 1**.

**Table 1:** Clinicodemographic patient characteristics according to the dataset.

	SNUBH				SNUH
	Training	Temporal test #1	Temporal test #2	Total	Geographic test
Demographics	127	20	12	159	35
Mean age (years)	61 ± 12	63 ± 13	67 ± 12	62 ± 12	61 ± 12
M/F ratio	61/66	12/8	6/6	79/80	19/16
MRI	174	40	12	226	35
1.5 T	114 (66%)	20 (50%)	4 (33%)	138 (61%)	–
3.0 T	60 (34%)	20 (50%)	8 (67%)	88 (69%)	35 (100%)
Primary cancer type					
Lung	98 (78%)	17 (85%)	11 (92%)	126 (79%)	28 (80%)
Melanoma	1 (1%)	–	–	1 (1%)	2 (6%)
Breast	17 (14%)	3 (15%)	–	20 (13%)	2 (6%)
Renal	3 (2%)	–	–	3 (2%)	–
Gastrointestinal	4 (3%)	–	1 (8%)	5 (3%)	–
Genitourinary	–	–	–	–	1 (3%)
Sarcoma	1 (1%)	–	–	1 (1%)	–
Thyroid	1 (1%)	–	–	1 (1%)	1 (3%)
Ovary	1 (1%)	–	–	1 (1%)	1 (3%)
Head and neck	1 (1%)	–	–	1 (1%)	–

MRI, magnetic resonance imaging; SNUBH, Seoul National University Bundang Hospital; SNUH, Seoul National University Hospital.

### Performance of CAD

#### Temporal Test Set #1

**Figure 3:**
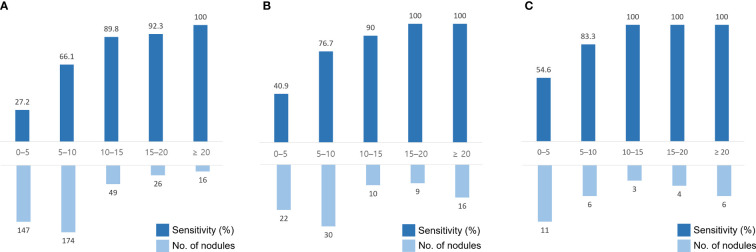
Sensitivity and number of brain metastases (BMs) in different nodule sizes in the temporal test set #1 **(A)**, the geographic test set **(B)**, and the temporal test set #2 **(C)**. The x-axis indicates the size of the nodules (mm).

The sensitivity for BM detection in the temporal test set was 58.0% (239 of 412; 95% confidence interval [CI], 53.2%–62.7%), the mean DSC was 0.67 ± 0.23, and the FP rate per scan was 2.5 (99/40). For BM measuring ≥ 5 mm, the sensitivity was 75.1% (199 of 265; 95% CI, 69.6%–79.9%), the mean DSC was 0.69 ± 0.22, and the FP rate per scan was 0.8 (33/40). The median BM volume was 30 mL (IQR, 8–119 mL). The sensitivity and number of BMs across the nodule size in each dataset are shown in **Figure 3**. For registration, all 69 BMs detected on serial MRI examinations were successfully matched.

#### Agreements in the RANO-BM Criteria

**Figure 4:**
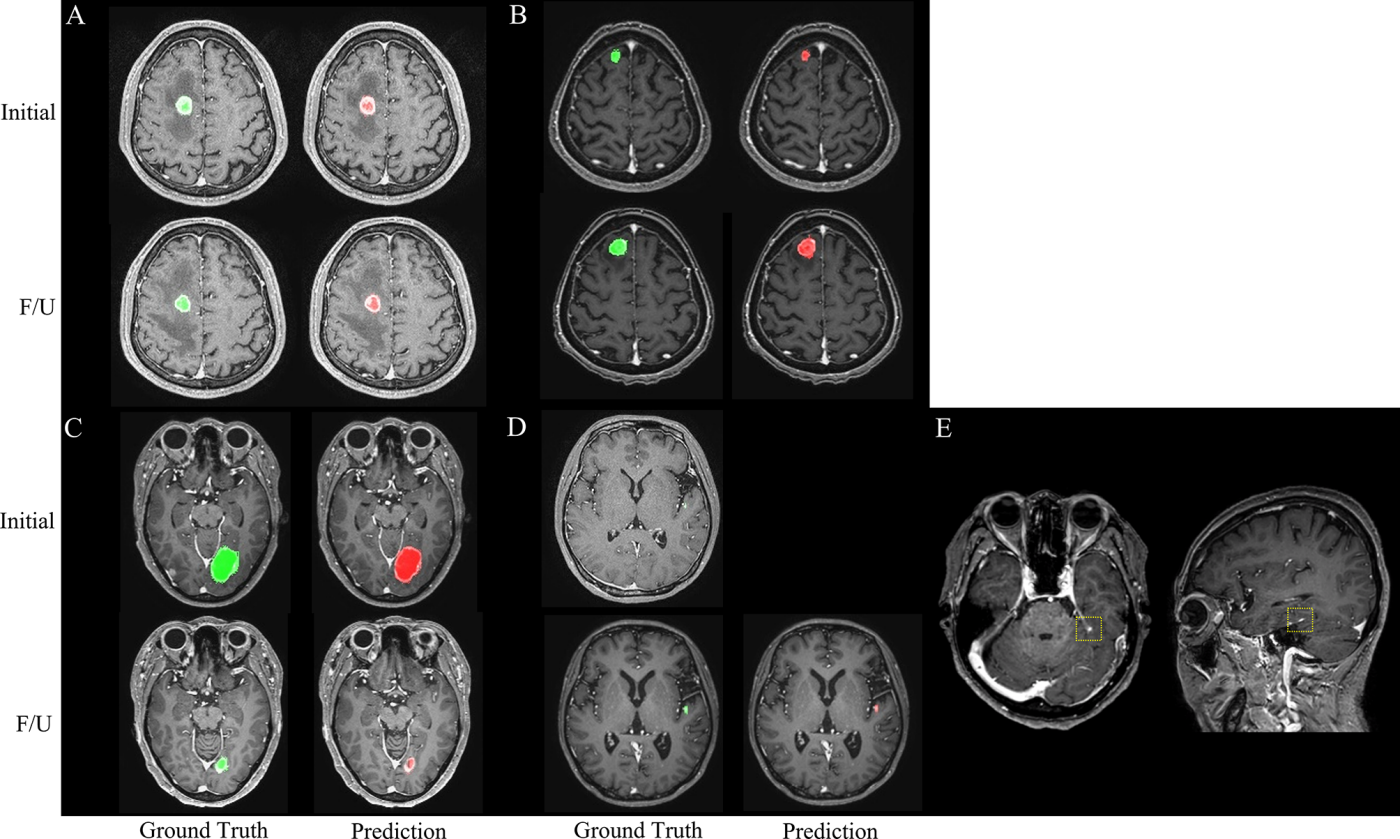
Representative cases **(A–E)** for automated brain metastasis (BM) detection, segmentation, and treatment response assessment using our proposed deep learning computer-aided detection (DL-CAD) system. **(A)** Stable disease. **(B)** Progressive disease. **(C)** Partial response. **(D)** False-negative detection of a small BM in the left temporal lobe on initial MRI, which showed enlargement and was correctly detected on a follow-up MRI. **(E)** False-positive detection. A small cortical vein was falsely detected by DL-CAD (dotted square), which could be easily differentiated by radiologists.

**Figure 5:**
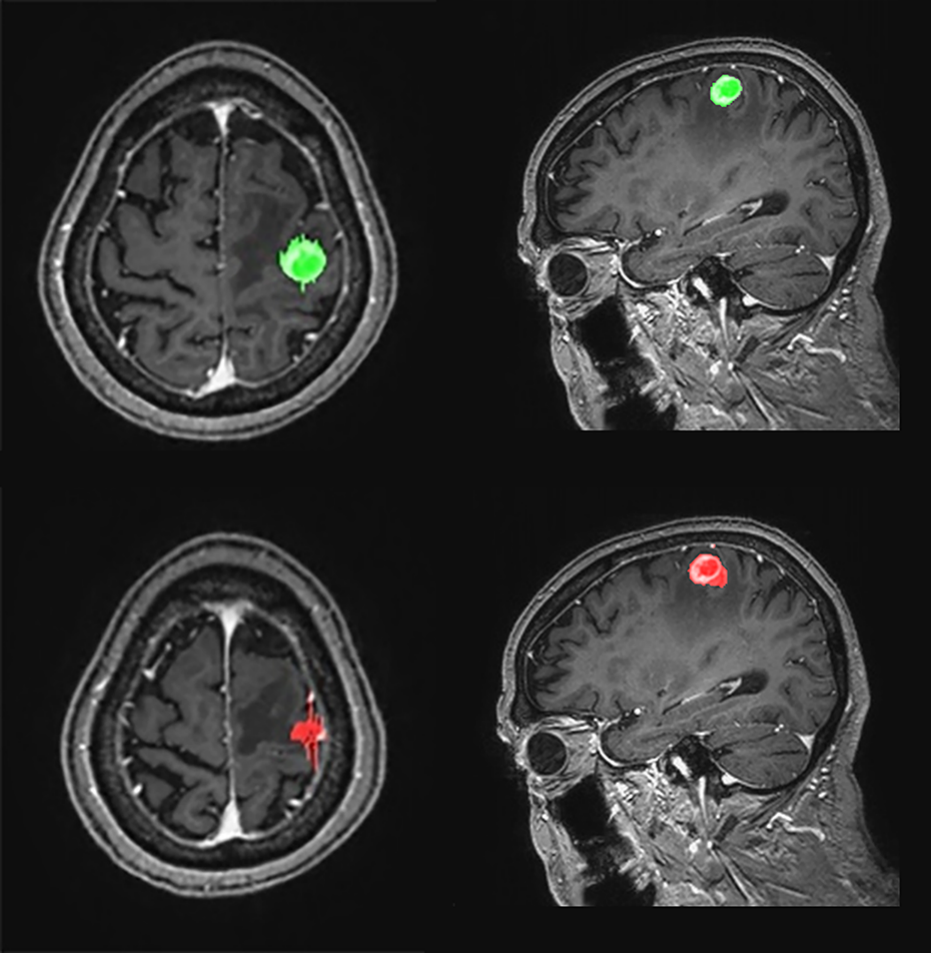
Graphical representation of one-dimensional and volumetric measurement of the ground truth (upper row) and deep learning computer-aided detection (DL-CAD) system (lower row). The longest diameter and volume of the ground truth was 19 mm and 1597 mL, respectively, whereas the longest diameter and volume measured by DL-CAD was 27 mm and 1590 mL, respectively. Thus, volumetric measurement showed better agreement between DL-CAD and the ground truth than one-dimensional measurement (Dice similarity coefficient was 0.85).

The agreements in the RANO-BM criteria between DL-CAD and ground truth assessed by experienced radiologists were moderate (**κ**, 0.52; 95% CI, 0.26–0.79) for one-dimensional measurement and substantial (**κ**, 0.68; 95% CI, 0.41–0.94) for volumetric measurement (**Table 2**). Representative cases are illustrated in **Figures 4** and **5**.

**Table 2:** Agreement in the response assessment in neuro-oncology brain metastases (RANO-BM) criteria.

	One-dimensional GT					Volumetric GT				
	CR	PR	SD	PD	Total	CR	PR	SD	PD	Total
**DL-CAD**										
CR	2	1	1	0	4	2	1	0	1	4
PR	0	3	3	2	8	0	3	1	1	5
SD	0	1	0	0	1	0	0	3	1	4
PD	0	0	1	6	7	0	0	0	7	7
Total	2	5	5	8	20	2	4	4	10	20

GT, ground truth; CR, complete response; PR, partial response; SD, stable disease; PD, progressive disease; DL-CAD, deep learning-based computer-aided detection system.

#### Geographic Test Set

Regarding the geographic test set, the sensitivity of detection was 75.9% (66 of 87; 95% CI, 65.5%–84.4%), the mean DSC was 0.66 ± 0.22, and the FP rate per scan was 7.6 (265/35). For BMs measuring ≥ 5 mm, the sensitivity was 87.7% (57 of 65; 95% CI, 77.2%–94.5%), the mean DSC was 0.68 ± 0.20, and the FP per scan was 1.9 (67/35). The median BM volume was 279 mL (IQR, 83–1629 mL).

#### Temporal Test Set #2

Regarding the temporal test set #2, the sensitivity of detection was 80.0% (24 of 30; 95% CI, 61.4%–92.3%), the mean DSC was 0.76 ± 0.26, and the FP rate per scan was 2.2 (26/12). For BMs measuring ≥ 5 mm, the sensitivity was 94.7% (18 of 19; 95% CI, 74.0%–99.9%), the mean DSC was 0.82 ± 0.20, and the FP per scan was 0.5 (6/12). The median BM volume was 353 mL (IQR, 65–2140 mL).

## Discussion

Accurate treatment response assessment for BM is crucial; however, it is highly labor intensive. Our proposed DL-CAD showed promising results for automated assessment of treatment response of BM. Using the modified RANO-BM size criteria for measurable disease, the detection sensitivity was 75.1%, 94.7%, and 88% for BMs measuring ≥ 5 mm in the temporal test #1, temporal test #2, and geographic test sets, respectively. In all cases, the automatic co-registration of detected lesions on serial MRIs (pre-treatment and post-treatment) was successful. Subsequent automated treatment response assessment showed a moderate degree of agreement for one-dimensional measurement, and substantial agreement for volumetric measurement between DL-CAD and experienced neuroradiologists.

As previously reported (24), the detection sensitivity of DL-CAD models primarily depends on the size of BM nodule. Small metastases with poorly defined boundaries and low contrast typically cause an inevitable increase of FP nodules detected on DL-CAD. However, the issue of impaired sensitivity due to small metastases was minimized by following the RANO-BM criteria, in which up to five target lesions were selected based on their size (26). In our study, the average sum of the longest diameters and volumes of five target lesions on the initial MRI in the testing set was 43.7 mm and 1859.6 mL per patient, respectively. We showed that automated quantitative analysis of MRI using a comprehensive DL approach could be a valuable tool for clinical decision making in neuro-oncology. As was demonstrated by Bauknecht et al. (27) automated treatment response assessment using the RANO-BM criteria is promising for lowering interobserver variability and improving individualized patient management. Although quantitative volumetric assessment of tumor response might arguably be one of the most quintessential parameters for accurate assessments of tumor burden and response (41–43), it has previously been cited as a labor-intensive, time-consuming, and complex task that ultimately prevents clinical adoption (44, 45). Our effort to evaluate the usefulness of a fully automated quantitative analysis of MRI in neuro-oncology showed that it has the potential to overcome the inherent limitations of manual assessment of tumor burden. We assume that suboptimal detection sensitivity and FP detection rate influenced the moderate agreement of RANO-BM assessment using one-dimensional measurement. A single, small FP nodule on follow-up MR images could lead to “progression” on RANO-BM assessment. In addition, inaccurate segmentation also could affect the accuracy of RANO-BM assessment due to inaccurate size measurement. However, the agreement of the RANO-BM criteria was higher for volumetric assessment than for one-dimensional assessment, indicating that the volumetric assessment could be reliably used to reduce the interobserver variability.

Compared with previously reported approaches using DL for assessing BM, our study has three major strengths. First, we showed the possibility of end-to-end automation of treatment response evaluation of BM, which is a tedious and time-consuming task for radiologists. Typically, radiologists follow several steps during evaluation of brain MRI in a patient with suspected BM: detection, comparison with prior images if available, followed by comparison of size changes. Most previous investigations (7–11, 24) have mainly focused on evaluating CAD in one or two steps such as detection or segmentation. However, similar to a recent study of end-to-end lung cancer screening (25) or brain tumor response (41), we showed the possibility of end-to-end evaluation of BM. Furthermore, these prior CAD studies typically report only a lesion-level performance. In contrast, our DL-CAD performed human-independent evaluation on full volumes. The excellent performance of registration of serial brain MRIs supported the possibility of end-to-end automated treatment response evaluation of BM. Second, to lower the selection bias and to enhance the generalizability of our results, we enrolled consecutive patients and used temporal separation of internal test data. In addition, we further performed validations using another temporally separated dataset and data from another institution. Cho et al. conducted a systematic review and meta-analysis of 12 studies on BM detection by machine learning (46). They found that only two studies included consecutive patients and conducted an external validation or temporal separation of test data. Therefore, the results of our study better depicted the real-world clinical setting than those of previous studies (7–11, 21, 22, 24). Third, we used the entire image context, therefore avoiding patch-wise inferences, which may lack robustness because of the broad range of BM sizes. The methodology proposed in this study has the advantage of dividing BM using two steps. In the first step, a 3D U-Net was used to locate small BMs in a large brain region. In the second step, the detected BM was cropped to avoid reducing the BM size in the image. In the cropped image, the DenseNet based U-Net model provides more accurate and detailed segmentation performance in the entire brain region than the direct segmentation of BM.

However, our study also has some limitations. First, we acknowledge the retrospective nature of the study and the relatively small, single-center dataset with both 1.5-T and 3.0-T MRI used for training of the DL-CAD, which may be insufficient for addressing the variabilities in scanning techniques and hardware implementations across hospitals. A larger training dataset from a multi-center study might allow further improvement of the accuracy of the DL-CAD. However, we used an external test set as well as a temporally separated internal test set to improve the generalizability of our results. In addition, we found a slightly higher detection sensitivity for the geographic test set and temporal test set #2 than that for the temporal test set #1. Second, although the system achieved a high sensitivity for larger metastases, it showed a limited detection performance for smaller metastases. Although we used a 3D U-Net CAD model based on the Dice loss function considering previous studies using the models specialized in detection such as RetinaNet (47) or YOLO (48), the system showed unsatisfactory detection sensitivity for small BM. However, Zhou et al. similarly reported a low sensitivity for smaller BM (24), in which their system showed 15% and 70% sensitivity for BM measuring ≤ 3 mm and ≤ 6 mm, respectively. Considering that BM ≤ 5 mm accounts for 35.7% of BMs in the internal testing set, consecutive enrollment in our study also might have led to a more difficult testing set. Recent imaging protocols in BM recommend that turbo-spin echo T1WI should be preferred over conventional 3D GRE T1WI (5). Therefore, larger future studies with black-blood imaging (49, 50) might be helpful for improving the detection sensitivity and reducing FP findings. Third, our DL-CAD has limitations in evaluating leptomeningeal seeding or skull metastasis. Fourth, this study did not include clinical components such as steroid use or neurological deterioration of patients during treatment response assessment. Finally, we chose operating points for the DL-CAD primarily to compare reader and model performance. It should be noted that the selection of operating points for use in clinical practice remains an ongoing area of research, potentially involving outcomes to properly trade-off between sensitivity and specificity. More robust retrospective and prospective studies would be required to ensure clinical applicability.

Our proposed DL-CAD showed the potential for automated treatment response assessment of BM lesions measuring ≥ 5 mm. These results represent a step toward automated BM evaluation *via* DL. We believe this research could supplement future approaches to BM evaluation as well as support assisted- or second-read workflows. In addition, we believe the general approach employed in our work, which mainly involved outcomes-based training, full-volume techniques, and directly comparable clinical performance evaluation, may lay additional groundwork toward DL medical applications.

## Data Availability Statement

The datasets presented in this article are not readily available because they are subject to the permission of the Institutional Review Boards of the participating institutions. Requests to access the datasets should be directed to leonard.sunwoo@gmail.com.

## Ethics Statement

The studies involving human participants were reviewed and approved by Institutional Review Boards of Seoul National University Bundang Hospital and Seoul National University Hospital. Written informed consent for participation was not required for this study in accordance with the national legislation and the institutional requirements. Written informed consent was not obtained from the individual(s) for the publication of any potentially identifiable images or data included in this article.

## Author Contributions

Conceptualization, LS. Methodology, YK, GL, and LS. Software, YK, GL, and KK. Validation, JC, LS, SC, YB, BC, CJ, JH, C-YK, C-HS, and JK. formal analysis, JC, YK, LS, and GL. Investigation, JC, YK, LS, and GL. Resources, LS and KK. Data curation, JC, YK, LS, GL, and TN. Writing—original draft preparation, JC, LS, SC, SB, YB, BC, CJ, C-HS, JH, and C-YK. Writing—review and editing, LS and KK. Visualization, JC, YK, LS, and GL. Supervision, LS and KK. Project administration, LS and KK. Funding acquisition, LS and KK. All authors contributed to the article andapproved the submitted version.

## Funding

This research was funded by the National Research Foundation of Korea (NRF-2018R1C1B6007917) and the Gachon Gil Medical Center (FRD2019-11-02(3)).

## Conflict of Interest

The authors declare that the research was conducted in the absence of any commercial or financial relationships that could be construed as a potential conflict of interest.

## Publisher’s Note

All claims expressed in this article are solely those of the authors and do not necessarily represent those of their affiliated organizations, or those of the publisher, the editors and the reviewers. Any product that may be evaluated in this article, or claim that may be made by its manufacturer, is not guaranteed or endorsed by the publisher.
